# Supplementary figures and images for: Genome-scale reconstructions to assess metabolic phylogeny and organism clustering
Source: PLoS One. 2020 Dec 29;15(12):e0240953. doi: 10.1371/journal.pone.0240953 (PMC7771690; doi:10.1371/journal.pone.0240953)

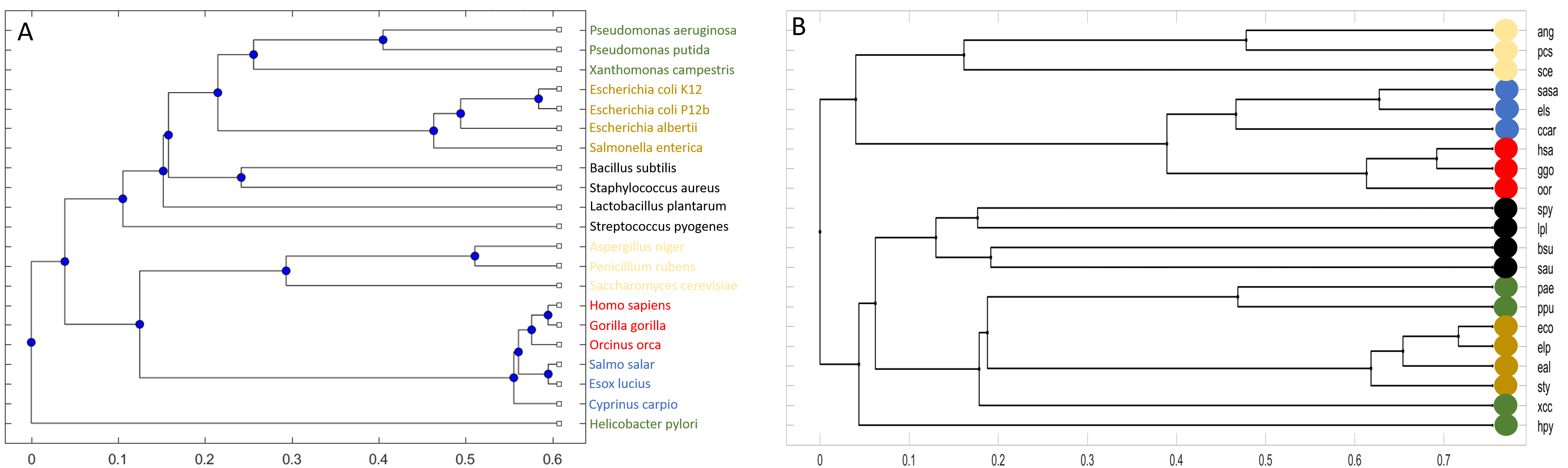

Supplement: S1 Fig — (TIF) [file pone.0240953.s009.tif]
